# Perceived Barriers of Clinical Roles Towards Intensive Care Unit Mobility

**DOI:** 10.1155/2024/5551184

**Published:** 2024-09-09

**Authors:** Hassan Y. Aljohani, Shahad Alammar, Shoug Alnawmasi, Riham Alfawzan, Nouf Alotaibi, Noora Mumenah, Arwa Alruwaili, Saleh S. Algrani, Tareq F. Alotaibi, Mobarak K. Alqahtani, Mohammed M. Alqahtani, Abdullah M. Alanazi, Taha Ismaeil, Sarah Almalki, Jassas Alotaibi

**Affiliations:** ^1^ Department of Respiratory Therapy King Saud bin Abdulaziz University for Health Sciences (KSAU-HS) & King Abdullah International Medical Research Centre (KAIMRC), Riyadh, Saudi Arabia; ^2^ Department of Physical Therapy Asir Rehabilitation Centre Asir Central Hospital, Abha, Saudi Arabia; ^3^ Physical Therapy Department King Fahad Medical Complex, Dhahran, Saudi Arabia

**Keywords:** attitude, barriers, behavior, healthcare providers, ICU, knowledge, mobility

## Abstract

**Background:** There is overwhelming evidence of improved patient outcomes as a result of early mobilization in the intensive care unit (ICU). However, several barriers of ICU mobility remain understated with reference to clinical roles. The purpose of this study is to investigate the perceived barriers of early mobility of critically ill patients among ICU healthcare providers.

**Methods:** In this cross-sectional study, the Mobilization Attitudes and Beliefs Survey (PMABS-ICU) was administered to ICU healthcare providers using an online survey. The study investigated barriers related to knowledge, attitudes, and behaviors regarding ICU mobility practices. These barriers were compared among different ICU clinical roles.

**Results:** The survey yielded a total number of 214 healthcare providers with 41% female and 59% male. Respiratory therapists reported the highest perceived barriers to ICU mobility (M 39, IQR 36, 43) % compared to physical therapists (who reported the lowest barriers), occupational therapists, nurses, and physicians (*p* ≤ 0.05). ICU healthcare providers' behavior towards ICU mobility such as perceived benefits and safety is ranked as the primary barrier (M 49, IQR 42, 52) %. Professional experience did not significantly vary among all groups.

**Conclusion:** Our findings highlight that ICU healthcare providers' perceptions, including both potential benefits and safety concerns regarding mobility, are significant barriers to implement mobility practices. ICU mobility barriers should be tackled by providing education and training. A focused effort to include RTs and nurses could advance interdisciplinary ICU mobility practice and reduce associated barriers.

## 1. Introduction

The number of patients in the United States who are admitted to the intensive care units (ICUs) reaches approximately 5.7 million annually [1]. Recent advances in ICU management have improved ICU clinical outcomes and survival rates for these patients [2]. However, many ICU patients develop negative outcomes as a result of bed rest and immobility such as ICU-acquired weakness [3–5]. Furthermore, ICU survivors report post-traumatic stress disorder, anxiety, depression, and other mental, cognitive, and physical impairments [1, 3, 6].

Early mobility is a safe and effective intervention that has been shown to improve muscular strength, functional independence, and mechanical ventilation duration use [2, 7, 8]. It is also associated with shorter ICU and hospital length of stay, lower incidence of delirium and mortality, greater hospital survival rates, as well as less time spent in rehabilitation [7, 9, 10]. Early mobility is a critical component of the ABCDEF bundle which is a multimodal evidence-based approach to the comprehensive treatment of critically ill patients, with the goal of optimizing patient recovery. The ABCDEF bundle refers to assessment, treatment, management of pain, spontaneous breathing trial and spontaneous awakening trial, choice of analgesia, management of delirium, and family engagement [9, 10].

Despite the benefits of ICU early mobility, several obstacles can impede its implementation by healthcare providers (HCPs), including limited personnel for early mobilization, lack of adequate training to facilitate mobility, shortage of time, and lack of equipment [11–13]. Early mobility engages multidisciplinary teams to provide mobility along the patient's hospitalization ensuring the safety of the care provided to patients [12]. Other obstacles remain during ICU mobility, including patient acuity, confusion about when to begin moving the patient, increased expenditures, lack of early mobilization protocols, and excessive sedation [10].

Assessing the status of rehabilitation services in the ICU is an important step to improve the ICU care provided in each country [14]. The current study investigated the perceived barriers among HCPs' clinical roles about early ICU mobility. The purpose of this study is to explore common barriers to ICU mobility to establish a baseline understanding and inform HCPs of current clinical practices.

## 2. Methods

This study was approved by the institutional review board of the King Abdullah International Medical Research Center (KAIMRC) (SP22R/077/05), Riyadh, Saudi Arabia. Written informed consent for the participation and publication of this study's findings was obtained from all participants. Data collection was conducted between September and November 2022. The study involved different disciplines including (respiratory therapists (RTs), nurses, physicians, occupational therapists (OTs), and physical therapists (PTs)) working at medical, cardiac, surgical, or pediatric ICUs in Saudi Arabia. HCPs who work in other than tertiary hospitals and other providers who do not have direct contact with patients in the ICU were excluded.

Participants were collected through convenient sampling and snowballing techniques. Independent study variables were years of experience, profession, and type of ICU. Moreover, online surveys with flyers have been used for data collection and were distributed by email for voluntary participation. The Mobilization Attitudes and Beliefs Survey ICU (PMABS-ICU) was used. The survey has been tested for validity and reliability with a Cronbach alpha ranging from 0.76 to 0.85 for internal consistency [15, 16]. The survey consists of 26 items. A 5-point Likert scale was used for response options (0 = *not applicable*; 1 = *strongly disagree*; 2 = *disagree*; 3 = *neutral*; 4 = *agree*; 5 = *strongly agree*). Open-ended questions (free text) are for any other comments on patient mobility concerns. The overall barriers scale and the three subscales (i.e., knowledge, attitude, and behavior) were scored on a scale of 0 to 100, with higher scores indicating more mobility barriers. Some items are reverse-scored (1 becomes 5 and vice versa) to reflect a more positive attitude towards mobility. Each subscale has its own sum based on the relevant items and its own possible score considering missing answers. The subscale scores are calculated by subtracting the sum of the scores from the maximum possible score for that subscale and then dividing by the maximum possible score and multiplying by 100.

The Statistical Package for the Social Sciences (SPSS) was used to conduct the analysis after the data was entered using Microsoft Excel. If the data were not normally distributed, median and interquartile ranges were used for continuous variables. Frequency and percentage were utilized for the categorical variables. We performed nonparametric tests to assess statistically significant differences between groups due to the non-normal distribution of our data. The chi-square test and the Kruskal–Wallis test were used. To identify specific group differences following a significant Kruskal–Wallis test result, a post hoc Dunn's test with the Bonferroni correction for multiple comparisons was conducted. A *p* value of less than 0.05 was considered statistically significant in all tests to identify significant overall differences in ICU mobility barriers across the rehabilitation groups.

## 3. Results

The majority of 214 participants were male HCPs. In addition, most of the participants work in the Central Region (51.9%). The median age of males and females was 28.5 years old (IQR 25, 35) years. Participants were recruited from different hospitals across the country with most participants working at the Ministry of National Guard or Ministry of Health (IQR 35.5, 26.6) %. HCPs were categorized into five ICU units, and the majority were working in the medical ICU (72.9%). The years of experience were approximately divided into three thirds: ≤ 2 years, 3–9 years, and ≥ 10 years. Moreover, 81.8% of participants had ICU mobility experience (Table 1).

**Figure 1:**
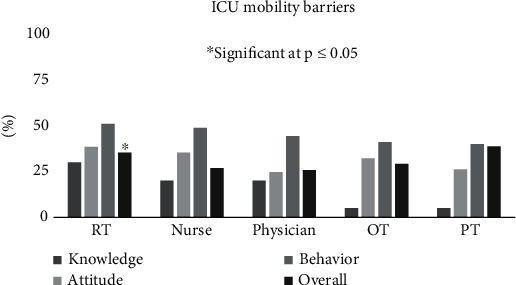
ICU mobility barriers encountered by health care providers among different specialties using three subscales (knowledge, attitude, and behavior).

Behavioral barriers were the highest for all disciplines with (M 49, IQR 42, 52) %. In addition, RTs significantly perceived the highest overall level of ICU mobility barriers, (M 39, IQR 36, 43) % *p* ≤ 0.05 compared to other disciplines. Furthermore, RTs had the highest knowledge barriers compared to OTs and PTs while physicians had the lowest perceived barriers for attitudes towards ICU mobility (Figure 1).

**Figure 2:**
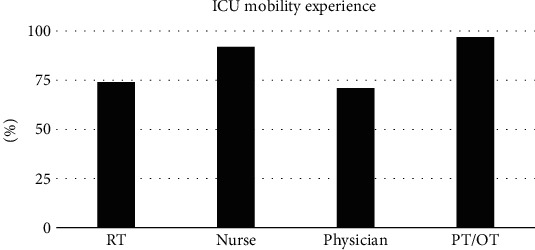
Healthcare providers' experience in mobilizing ICU patients.

OTs and PTs had the highest percentage of experience in mobilizing ICU patients (100%, 94%) and nurses followed with the percentage of (91.9%). On the other hand, RTs and physicians had the lowest ICU mobility experience (73.3%, 71%) (Figure 2).

Also, shortage of staff, having no protocols or guidelines, insufficient time, sedation, family involvement, and patient rejection were the main reported barriers by the participants in the open-ended section of the questionnaire.

## 4. Discussion

This study investigated the perceptions of HCPs about ICU early mobility barriers. The study has shown various barriers for ICU early mobilization categorized into three behavioral, knowledge, and attitudinal barriers. Behavioral barriers were found to be the highest among all disciplines.

The challenge of raising awareness and providing adequate training for critical care rehabilitation is a global issue [17]. The implementation of early mobilization protocols in the ICU can be challenging for a variety of factors, including low confidence due to a lack of staff training and insufficient resources [18]. According to the survey responses, behavioral barriers were often related to safety concerns. Many participants held the mistaken belief that patients were too sick to be mobilized and that increasing mobilization could harm patients and worsen their outcomes, leading to concerns about safety and fear of adverse events [19]. Also, participants believed that mobility would entail additional work responsibilities and did not feel confident in their ability to mobilize patients, believing that OTs and PTs should be the primary HCPs responsible for patient mobilization. Multiple ICU rehabilitation studies have emphasized the importance of cooperation among ICU multidisciplinary team members by creating designated champions to provide coordinated team effort and comprehensive strategy for safe and effective ICU mobilization [20, 21]. In addition, making appropriate referrals to other relevant clinical disciplines is crucial for successfully improving the outcomes of ICU survivorship [22].

One possible explanation for the higher level of barriers observed in this study is the lack of adequate protocols and targeted ICU mobilization programs for HCPs [17, 18]. Previous research has shown that most HCPs identified the absence of written guidelines and adequate training as major barriers to early mobility [11, 23]. In Saudi Arabia where this study took place, it has been reported that ICU mobility is implemented in 47% of ICUs. However, among the surveyed ICUs, 64% stated that their staff had not received training on how to mobilize ICU patients, and 55% reported the absence of ICU mobility protocols [24]. As such, it is crucial to develop ICU early mobility protocols and training programs that support current clinical needs for safe ICU mobilization and promote interdisciplinary practice [25]. Training programs and protocols should be based on the best available evidence and tailored to the specific needs and characteristics of each ICU setting. They should also involve a multidisciplinary team approach and address the potential barriers and facilitators for early mobilization [11]. Overcoming this challenge and emphasizing the importance of implementing rehabilitation protocols from the first day of ICU admission are crucial for driving a change in ICU culture [17].

Our study also found that PTs and OTs, who identified themselves to have the most mobility experience, perceived the lowest mobility barriers, while RTs significantly perceived the highest overall level of ICU mobility barriers compared to other disciplines. This suggests that higher ICU mobility experience could be a factor for lower ICU mobility barriers. Similarly, previous research found that the mobilization capacity for nurses and physicians was significantly lower capacity than physical therapists [26, 27].

Our findings highlight a critical need to address barriers to ICU mobility practices among HCPs in Saudi Arabia. The high prevalence of barriers observed in this study, coupled with the low reported rates of ICU mobility training and protocol implementation [28], suggests significant room for improvement. This aligns with Saudi Arabia's Vision 2030, a strategic plan to enhance the country's healthcare system and improve quality of life [29]. Integrating early mobilization and critical care rehabilitation principles into medical and health school curriculums can equip future graduates with the knowledge and confidence to implement these practices effectively [30].

Hospitals as well as official bodies that regulate health-care-related practices and training such as the Saudi Commission for Health Specialties (SCHS) may also recognize continuing education programs on early mobilization. These programs should be designed to offer hands-on workshops and simulations to build practical skills [19]. Such programs may include intensive training focused on theoretical knowledge and practical skills, workshops, and regular interdisciplinary meetings. Hospitals may also promote the use of standardized protocols and educate and involve patients and families in the mobilization process [9, 19, 26].

Although our research included different disciplines of RTs, nurses, physicians, OTs, and PTs from different healthcare sectors and regions within Saudi Arabia, future research should aim for a higher number of sample size to have more generalizable data. While the sample was likely to be representative of RTs, comprising 42% of the total, the remaining 58% was distributed among other clinical roles, potentially indicating an uneven distribution. This could be considered a limitation of the study.

Our study showed that behavioral factors, such as perceived benefits and safety concerns, were the most significant barriers to ICU mobility among HCPs. To address these barriers, it is essential to provide ICU staff with comprehensive training programs and protocols that facilitate the delivery of safe and effective early mobilization interventions [28]. These programs should be evidence-based and tailored to the specific needs and characteristics of each ICU setting, incorporating a multidisciplinary team approach and addressing potential barriers and facilitators for early mobilization (28).

Although our research included different disciplines of RTs, nurses, physicians, OTs, and PTs from different healthcare sectors and regions within Saudi Arabia, future research should aim for higher number of sample size to have more generalizable data. While the sample was likely to be representative of RTs, comprising 42% of the total, the remaining 58% was distributed among other clinical roles, potentially indicating an uneven distribution. This could be considered a limitation of the study.

In conclusion, the study indicates that behavioral factors, such as perceived benefits and safety concerns, were the most significant barriers to ICU mobility among HCPs. To address these barriers, it is essential to provide ICU staff with comprehensive training programs and protocols that facilitate the delivery of safe and effective early mobilization interventions. These programs should be evidence-based and tailored to the specific needs and characteristics of each ICU setting, incorporating a multidisciplinary team approach and addressing potential barriers and facilitators for early mobilization.

## Figures and Tables

**Table 1:** Demographic data of participants that includes gender, type of hospitals, regions, type of ICUs, clinical roles, years of work experience, and ICU mobility experiences (*N* = 214).

**Variable**	**N** ** (%)**
Gender	
Male	127 (59.3)
Female	87 (40.7)
Type of ICUs^a^	
Medical	156 (72.9)
Surgical	105 (49.1)
Cardiac	77 (36)
Pediatric	60 (28)
Clinical Roles	
Respiratory therapist	90 (42)
Physical therapist/occupational therapist	56 (26)
Nurse	37 (17.3)
Physician	31 (14.5)
Work experience (years)	
≤ 2 years	80 (37.4)
3–9 years	73 (34.1)
≥ 10 years	61 (28.5)
ICU mobility experiences	
Yes	175 (81.8)
No	39 (18.2)

^a^Some HCPs work at more than one type of ICU; therefore, percentages reveal a total of more than 100%.

## Data Availability

Data used to support the findings of this study are available from the corresponding author upon request.
